# Interventional Radiology Society of Australasia (IRSA) White Paper on Clinical Practice in Interventional Radiology

**DOI:** 10.1007/s00270-025-04264-8

**Published:** 2025-11-05

**Authors:** Warren Clements, Brendan T. Buckley, Radha Popuri, Philip Chan, Caitlin C. Farmer, Matthys van Wyk, Christopher Rogan

**Affiliations:** Interventional Radiology Society of Australasia, P.O.Box 1813, Sydney, NSW 2035 Australia

**Keywords:** Clinical, Practice, White paper, IR, Embolisation

Interventional Radiology Society of Australasia (IRSA) is a not-for-profit member society governed by the executive committee. IRSA is the peak membership body for healthcare professionals working in the field of interventional radiology in Australia and New Zealand. Providing continuing education, clinical practice resources, and collaboration opportunities for its members, IRSA advances awareness of IR with other healthcare providers and patients. IRSA works collaboratively with other stakeholders including the Royal Australian and New Zealand College of Radiologists (RANZCR) and is an affiliate member of the Cardiovascular and Interventional Society of Europe (CIRSE).

Medical imaging forms a core component of health networks, providing essential services to all hospital units. Medical imaging may be used for diagnosis or image-guided therapy. The dichotomy between diagnostic radiology (DR) and interventional radiology (IR) has resulted in a progressive and distinct clinical and strategic direction for IR since its inception in the 1960s [1].

Over the last decade, IR’s role has evolved from a technical service delivery model to IR clinician-led provision of comprehensive patient care, with complete clinical care at its core [2]. This is necessary to provide an optimal longitudinal model of care for patients, from referral through treatment and follow-up, and has been detailed in the 2021 CIRSE clinical practice manual as well as several publications [2, 3]. This aligns with existing models of specialist care in other medical and surgical specialties.

IR has been recognised as a formal specialty or subspecialty in many countries, including; the USA, UK, Japan, Portugal, and Brazil. RANZCR, with the support and assistance of IRSA, has been working towards specialty status for IR in Australia and New Zealand. The RANZCR interventional radiology training programme specifies clinical practice and competence within its core learning outcomes, is integrated into each of the structured learning domains, and features within programmatic assessment [4]. This includes outpatient clinic consultation, ward-based care, and multidisciplinary management, leading to longitudinal patient-focussed IR care. Clinical practice also forms key components of the RANZCR grandparenting programme and future training site accreditation [4]. In addition to training, the RANZCR standards of practice for interventional radiology outline the minimum standards that individuals and healthcare sites should adhere to when providing IR longitudinal care to patients [4]. Most importantly, it is a requirement that patients have access to direct consultation with their treating IR clinician, to ask questions, and to ensure that the care being provided meets international benchmarks for quality and accountability. This extends beyond the physical performance of a particular treatment alone [5].

IRSA recommends the provision of direct patient-facing clinical care in line with the standards set out by RANZCR and CIRSE. To ensure interventional radiologists can better serve the Australian and New Zealand patient population, IRSA has detailed additional guidance on the clinical component of care beyond RANZCR standards, to ensure that local hospital governance meets the expectations and requirements of training and practice standards. These recommendations will allow healthcare settings to understand how they can support and foster IR clinical practice. IRSA believes that these recommendations provide for patient-centred longitudinal care and will lead to improved quality of care and patient outcomes.

In 2025, the IRSA executive developed this white paper in accordance with the RANZCR Standards of Practice [4] and CIRSE clinical practice manual, in line with the IRSA constitution. This was developed via a modified and truncated Delphi-style process firstly following a face to face meeting, and with 2 rounds of email voting of the draft recommendations. On 19/08/2025, a draft document was circulated to the IRSA membership via email, and 2 weeks of member consultation was provided. The final document incorporates member feedback and has unanimous IRSA executive approval. These finalised recommendations are outlined in Table 1.

**Table 1:** IRSA white paper recommendations

IRSA recommendation	Description
Recommendation 1	IRSA recommends the allocation of full clinical practice privileges to interventional radiologists, including individual hospital admission rights and allocation of hospital beds
Recommendation 2	IRSA recommends the development of an appropriate team of medical staff (including junior medical staff and nurse specialists) consistent with other services providing complete patient care. This team should grow proportionate to workflow. This statement aligns with standard 6 of the RANZCR standards of practice
Recommendation 3	IRSA recommends unfettered access allowing direct clinical referral to interventional radiology, both within the hospital (clinical services, emergency, ICU, etc.) and external to the hospital, including primary care (general practice) Clinical referrals to IR should be through the same mechanism used by services referring to other clinical specialties and separate to the RIS-PACS system used for ordering routine diagnostic imaging. Adequate access to full electronic medical records should be provided. The systems should allow referring doctors to clearly distinguish between a referral for diagnostic imaging and a referral for clinical consultation by an IR. As with standard clinical referrals, it will allow doctors to provide the relevant background and clinical information, allowing the IR to further assess and provide advice and/or treatment if necessary. IRs should ensure appropriate clinical documentation is made in the patients’ permanent medical record
Recommendation 4	IRSA recommends access to dedicated, adequately resourced specialist outpatient consulting clinics for interventional radiologists, with the ability to consult with patients referred both internally and externally to any hospital or health network. This aligns with standard 5 of the RANZCR standards of practice. This provides patients and IRs with a safe and suitable environment for comprehensive clinical review. This is critical to allow patients to consider what is best for them and where applicable, provide consent in a suitable environment. This will also allow time for multidisciplinary input, as necessary, requesting of appropriate investigations, documentation of clinical notes, and communication to the referring doctor. The clinic environment should be similar to those already established for other specialist clinical services in the healthcare setting
Recommendation 5	IRSA recommends workflow and key performance metrics that reflect quality clinical practice for interventional radiologists, which differ from those used for diagnostic reporting. This includes clinical sessions for outpatient clinic, inpatient ward rounds, review and processing of referrals, comprehensive documentation, quality assurance activities, and interdisciplinary communication such as consultation and MDT. This is essential for quality patient management and reduces clinical and institutional risk
Recommendation 6	IRSA recommends the option to form an interventional radiology department/service, independent of diagnostic radiology. Ideally, this should include the appointment of an independent IR department director. For IR services integrated within a DR department, IRSA recommends a governance structure that ensures that IR workflow is overseen by an independent IR clinical lead, in charge of strategic and resource planning to provide a quality interventional radiology service. Governance decisions regarding IR service provision should only be made following adequate consultation with the IR clinical lead. This statement aligns with standards 1 and 4 of the RANZCR standards of practice
Recommendation 7	IRSA recommends a model where interventional radiologists govern IR related capital equipment and consumables, relevant to their clinical practice. Equipment and stock should be up-to-date, safe, and relevant for the type of work they are performing. This requires reliable access to single-plane interventional radiology unit(s) relevant to the acuity workflow, and with a working life cycle agreed at the time of installation. For the majority of IR services, there should be 24-h access to the interventional radiology unit. An IR service should have access to dedicated interventional CT sessions, distinct from diagnostic CT provision. Combined angiography-CT suites are increasingly becoming a new standard of care, especially in the provision of advanced interventional treatment including interventional oncology IRSA believes that IRs should operate using dedicated interventional radiology units. Routine use of biplane angiography units, cardiac catheterisation laboratories, or C arm units is undesirable because these laboratories are purpose-designed for different situations and may not lead to best practice in patient flow, radiation protection, occupational health and safety standards, and thus optimal patient outcomes. This aligns with standards 2 and 3 of the RANZCR standards of practice

*IRSA* Interventional Radiology Society of Australasia, *RANZCR* Royal Australian and New Zealand College of Radiologists

While not the target of these recommendations, it is important that IRSA acknowledges the role of clinical radiologists (also known as diagnostic radiologists) who perform many image-guided procedures as a part of their scope of practice but without a direct patient-facing model of care. This cohort of clinical radiologists is also known as procedural radiologists by RANZCR. IRSA supports procedural radiologists in performing procedures according to the relevant RANZCR standards of practice and their institutional scope of practice.

In conclusion, interventional radiology training incorporates clinical practice and longitudinal patient care at its core, and this is underpinned by standards of practice and guidelines throughout the Australia/New Zealand region, and also worldwide. The statements in Table 1 highlight practical recommendations which local health networks should incorporate in order to meet the standards as set out by major regulatory bodies including RANZCR and CIRSE, and also which are expected by our patients in order to provide the highest quality of care from this specialty.

## Data Availability

Not applicable.
